# Chikungunya Virus Neutralization Antigens and Direct Cell-to-Cell Transmission Are Revealed by Human Antibody-Escape Mutants

**DOI:** 10.1371/journal.ppat.1002390

**Published:** 2011-12-01

**Authors:** Chia Yin Lee, Yiu-Wing Kam, Jan Fric, Benoit Malleret, Esther G. L. Koh, Celine Prakash, Wen Huang, Wendy W. L. Lee, Cui Lin, Raymond T. P. Lin, Laurent Renia, Cheng-I Wang, Lisa F. P. Ng, Lucile Warter

**Affiliations:** 1 Singapore Immunology Network, BMSI, A*STAR, Singapore; 2 National Public Health Laboratory, Ministry of Health, Singapore; 3 Department of Biochemistry, Yong Loo Lin School of Medicine, National University of Singapore, Singapore; University of North Carolina at Chapel Hill, United States of America

## Abstract

Chikungunya virus (CHIKV) is an alphavirus responsible for numerous epidemics throughout Africa and Asia, causing infectious arthritis and reportedly linked with fatal infections in newborns and elderly. Previous studies in animal models indicate that humoral immunity can protect against CHIKV infection, but despite the potential efficacy of B-cell-driven intervention strategies, there are no virus-specific vaccines or therapies currently available. In addition, CHIKV has been reported to elicit long-lasting virus-specific IgM in humans, and to establish long-term persistence in non-human primates, suggesting that the virus might evade immune defenses to establish chronic infections in man. However, the mechanisms of immune evasion potentially employed by CHIKV remain uncharacterized. We previously described two human monoclonal antibodies that potently neutralize CHIKV infection. In the current report, we have characterized CHIKV mutants that escape antibody-dependent neutralization to identify the CHIKV E2 domain B and fusion loop “groove” as the primary determinants of CHIKV interaction with these antibodies. Furthermore, for the first time, we have also demonstrated direct CHIKV cell-to-cell transmission, as a mechanism that involves the E2 domain A and that is associated with viral resistance to antibody-dependent neutralization. Identification of CHIKV sub-domains that are associated with human protective immunity, will pave the way for the development of CHIKV-specific sub-domain vaccination strategies. Moreover, the clear demonstration of CHIKV cell-to-cell transmission and its possible role in the establishment of CHIKV persistence, will also inform the development of future anti-viral interventions. These data shed new light on CHIKV-host interactions that will help to combat human CHIKV infection and inform future studies of CHIKV pathogenesis.

## Author Summary

Chikungunya virus (CHIKV) is transmitted by mosquito bites and causes a febrile disease that is often characterized by persistent joint pain. Until recently, CHIKV outbreaks were limited to tropical areas of Africa and Asia. However, since 2007, following a large CHIKV epidemic in the Indian Ocean and South-East Asia, CHIKV has also been reported in temperate European regions. As mosquito habitats expand, virus dissemination may become more prevalent, but there are currently no vaccines or CHIKV-specific treatments available. We previously described two human antibodies that potently block cellular CHIKV infection. In the current report, we have characterized CHIKV mutants that escape neutralization to identify sub-domains of the virus envelope which are involved in CHIKV interaction with these antibodies, thereby opening the door for the development of CHIKV-specific sub-domain vaccination strategies. For the first time, we have also demonstrated that CHIKV can be directly transmitted between cells, bypassing transport through the extra-cellular space. This mode of dissemination, which is associated with viral resistance to antibody neutralization, may play a critical role in the establishment of persistent CHIKV infection. Together, these findings will aid the design of new strategies to combat CHIKV infection and will inform future studies of CHIKV pathogenesis.

## Introduction

Chikungunya virus (CHIKV) belongs to the *alphavirus* genus of the *Togaviridae* family and is transmitted to humans by *Aedes* mosquitoes. CHIKV was first isolated in Tanzania in 1952 [1], with numerous outbreaks subsequently being reported throughout Africa and Asia. Within the last decade, a large CHIKV epidemic has spread from the Indian Ocean islands to India and South-East Asia [2], [3]. Moreover, cases of CHIKV infection have since been detected both in Italy, in 2007 [4], [5], and in France, in 2010 [6], indicating that CHIKV has now become an infectious threat that is no longer limited to tropical areas.

While CHIKV infection in humans is often associated with only mild clinical symptoms that resolve over 1–2 weeks [7], there have also been reports of prolonged joint pain [8], [9], active and destructive rheumatoid arthritis [10], and severe encephalopathic events in neonates [11]. Despite the increasing burden of infection in Africa and Asia, and the recent advance of CHIKV into European territories, specific therapies for CHIKV-infected patients are not yet available [12].

CHIKV exhibits a positive strand RNA genome that encodes 4 non-structural proteins (NSP1–4) and 5 structural proteins: the capsid (C), the E1, E2, and E3 envelope glycoproteins (E2 and E3 are initially synthesized as a single precursor molecule, p62, which is subsequently cleaved), and a small polypeptide molecule, 6K [13]. However, the mature CHIKV virion is comprised only of the C, E1 and E2 proteins, which encapsulate the virus genome [13]–[15]. The E1 and E2 proteins control viral entry into host cells: E1 mediates virus fusion to cell membranes in low pH conditions [16], [17], while E2 interacts with a cellular receptor [18], [19]. These constituent proteins of CHIKV virion mediate virus dissemination, therefore specific targeting of these structures will be key to the future development of effective CHIKV vaccination strategies.

The structure of the E1 protein in alphaviruses has previously been determined using the representative Semliki Forest virus family member [20], [21]. More recently, the crystal structure of the E1/E2 heterodimer in alphaviruses has also been resolved, both under neutral pH conditions (using CHIKV: [22]), and at acidic pH (using Sindbis virus: [23]) thus further clarifying the structural composition of the alphaviruses. The alphavirus E1 ectodomain comprises three separate sub-domains; the N-terminal domain I (central to the 3D structure of E1), the domain II (located at the distal tip of the ectodomain) containing the fusion peptide residues 83–98, and the C-terminal domain III (located close to the viral membrane). The E2 ectodomain also contains three distinct sub-domains; the N-terminal domain A (central to the 3D structure of E2), the domain B - located at the distal tip of the ectodomain and that may interact with a cellular receptor, and the C-terminal domain C (located close to the viral membrane).

Human antibodies isolated from the plasma of a CHIKV convalescent patient have previously been shown to both prevent and cure CHIKV infection in mice [24], suggesting that neutralizing antibody responses might be capable of efficiently controlling CHIKV infection in humans. Although the brief viremia associated with CHIKV infections is suggestive of rapid viral clearance, several recent reports have instead detected long-lasting CHIKV-specific IgM, suggesting that viral antigens may in fact persist in humans [25], [26]. Moreover, long-term survival of CHIKV in macrophages has also been reported in non-human primates [27], indicating that CHIKV might establish chronic infections that evade immune defenses.

Viruses can escape neutralizing antibody responses by undergoing genetic mutations that abolish antibody binding, or by indirect evasion strategies such as cell-to-cell transmission. It is currently unclear whether CHIKV is capable of exploiting these strategies to persist in human hosts.

In this study, we aimed to characterize CHIKV antigens targeted by neutralizing human antibodies to inform the subsequent design of CHIKV-specific sub-domain vaccination strategies. We also sought to identify potential mechanisms of immune evasion that CHIKV might exploit to establish persistent infections in man. We recently identified two human monoclonal antibodies (mAb), designated 5F10 and 8B10, which broadly and potently neutralize CHIKV *in vitro* [28]. In the current report, we analyzed neutralization-resistant CHIKV mutants to identify the E2 domain B and the fusion loop “groove” as the primary determinants of CHIKV interaction with 5F10 and 8B10, respectively. Furthermore, we provide evidence that CHIKV can be efficiently transmitted from cell to cell in a manner that depends on E2 domain A, and that is associated with resistance to antibody-dependent neutralization. Taken together, these data advance our understanding of CHIKV-human host interactions and will inform future efforts to combat this viral disease.

## Results

### Selection of CHIKV mutants escaping antibody-dependent neutralization

**Figure 1:**
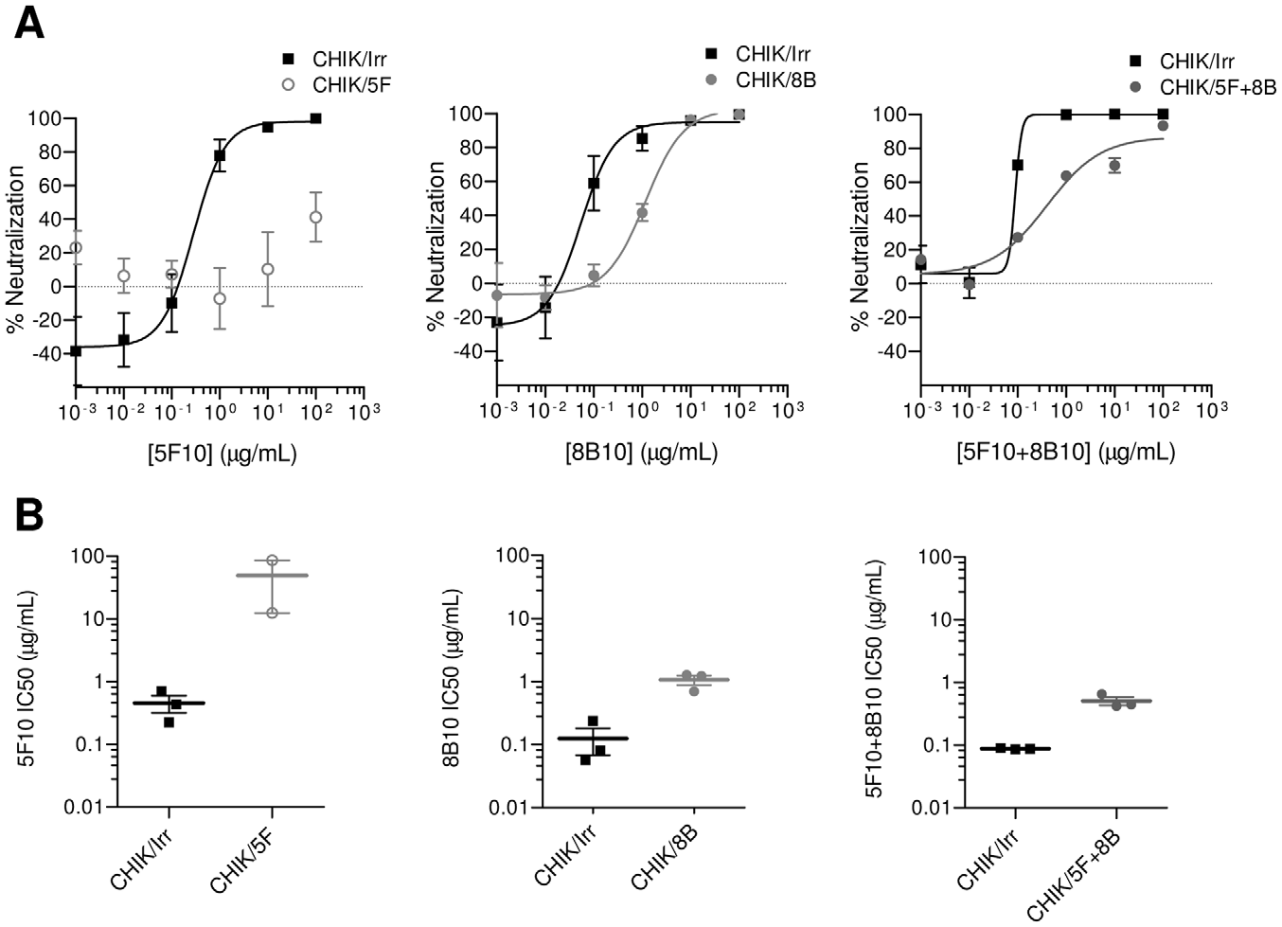
Reduced neutralizing potency of mAb 5F10 and 8B10 against CHIKVs amplified under selective pressure. CHIK/Irr, CHIK/5F, CHIK/8B and CHIK/5F+8B indicate CHIKV rescued from 8 serial cell passages under the continuous mAb pressure of Irr.IgG1, 5F10, 8B10 and 5F10+8B10, respectively. (**A**) The neutralizing potency of CHIKV-specific mAb was evaluated in PRNT over a concentration range of 1 ng-100 µg/ml. Displayed are the mean and SEM from 3 independent experiments performed in duplicate, with non-linear regression fitting curves. Regression could not be calculated for CHIK/5F due to non convergence. (**B**) The extrapolated IC50 from 2 or 3 independent experiments are shown, alongside the mean and SEM.

We recently identified two human mAb designated 5F10 and 8B10, which broadly and potently neutralize CHIKV *in vitro* (28). In order to identify the neutralization antigens that are targeted by these mAb, we subjected a CHIKV clinical isolate (CHIKV11) to 8 rounds of amplification under the continuous neutralizing pressure of mAb 5F10, 8B10, 5F10+8B10, or an irrelevant isotype-matched control (Irr.IgG1), before isolating viruses CHIK/5F, CHIK/8B, CHIK/5F+8B, and CHIK/Irr, respectively. When assessed in a Plaque Reduction Neutralization Test (PRNT), CHIK/Irr was efficiently neutralized by 5F10 and 8B10, either by each mAb independently, or by both mAb used in combination, whereas CHIK/5F was not neutralized by 5F10 (Figure 1). CHIK/8B and CHIK/5F+8B were less efficiently neutralized by 8B10 and 5F10+8B10 respectively than the CHIK/Irr control (Figure 1). These data indicated that CHIK/5F was resistant to 5F10 neutralizing activity, while CHIK/8B and CHIK/5F+8B were partially resistant to 8B10 and 5F10+8B10 neutralizing mAb.

To investigate whether CHIKV resistance to mAb 5F10 and/or 8B10 was associated with specific mutation(s), viral RNA was isolated from each CHIKV mutant and reverse-transcribed into cDNA for sequencing. When compared with the CHIK/Irr control genome, we identified two nucleotide (nt) substitutions within the CHIK/5F genome that resulted in amino acid (aa) changes at positions 82 and 216 in the E2 protein (E2.R82G and E2.V216E). In the CHIK/8B genome, we identified one nt substitution that resulted in an aa change at position 101 in the E1 protein (E1.T101M), and also observed 1 mix of wild-type (wt)/mutated nt, with the mutated nt leading to one *aa* change at position 12 of E2 (E2.T12I). We detected only one nt substitution in the CHIK/5F+8B genome, which generated the same E2.R82G substitution as previously identified in CHIK/5F genome (Table 1). We did not identify any mutations within the C, E3 or 6K protein-encoding sequences.

**Table 1:** Amino acid variations in the E1 and E2 glycoproteins among CHIKV variants amplified over 8 rounds under mAb pressure.

CHIKVs	E1^a^	E2^b^		
	101	12	82	216
CHIK/Irr	T	T	R	V
CHIK/5F	T	T	**G**	**E**
CHIK/8B	**M**	**T/I**	R	V
CHIK/5F+8B	T	T	**G**	V

a,b Numbers refer to the aa positions within the E1 and E2 CHIKV proteins, respectively. The aa variations associated with the CHIKV-specific mAb are highlighted in bold.

Intriguingly, the mutated nts associated with the residue substitutions E1.T101M, E2.T12I, E2.R82G and E2.V216E were also detectable in the polyclonal CHIK/Irr control cDNA preparation (Table 2), although their proportion was extremely low (0.05–0.20% of the total nts at each position). This suggests that minor pre-existing CHIKV quasi-species were amplified under selective pressure from CHIKV-neutralizing mAb.

**Table 2:** Next Generation Sequencing of non-clonal CHIK/Irr after 8 rounds under mAb pressure.

aa substitution^a^	nt substitution^b^	number of reads^c^	nt (%)^d^			
			A	T	C	G
E2.T12I	C1010T	7907	0.10	**0.08**	99.71	0.11
E2.R82G	A1219G	7826	99.58	0.04	0.18	**0.20**
E2.V216E	T1622A	7840	**0.05**	99.55	0.31	0.09
E1.T101M	C2729T	7999	0.14	**0.09**	99.76	0.01

a Numbers refer to the aa position within the indicated protein, E1 or E2.

b Nt substitutions associated with aa substitutions indicated in ^a^.

Numbers refer to the nt position within the C-E1 encoding sequence.

c Indicates the number of sequenced viral cDNA copies, as performed by Next Generation Sequencing.

d Indicates the percentage of each nt identified at each position.

### Key amino acid substitutions in the E1 and E2 proteins that confer CHIKV resistance to antibody-dependent neutralization

To investigate the roles of the different CHIKV mutations in mediating resistance to mAb 5F10 and 8B10, we isolated CHIKV clones that exhibited either single or dual mutations and then further probed their sensitivity to mAb-dependent neutralization.

CHIKV populations were cultured under agarose-medium before 6 individual CHIK/5F, CHIK/8B, or CHIK/5F+8B colonies (or 2 CHIK/Irr control colonies) were isolated for amplification and sequencing. The sequencing data from the plaque-purified CHIKV clones are shown in Table 3. The 6 CHIK/5F-derived clones contained both the E2.R82G and E2.V216E mutations. One of the CHIK/5F-derived clones also contained 2 additional aa substitutions within the E1 trans-membrane domain (T396A and I427T). Five of the CHIK/8B-derived clones contained both the E1.T101M and E2.T12I mutations, while another CHIK/8B-derived clone contained the E1.T101M mutation alone. All 6 CHIK/5F+8B-derived clones contained the E2.R82G mutation. Hereafter, the 5F10-derived clonal viruses are designated 5F/E2.R82G+V216E and 5F/E1.TMm+E2.R82G+V216E, the 8B10-derived virus mutants as 8B/E1.T101M and 8B/E1.T101M+E2.T12I, and the 5F10+8B10-derived virus, as 5F+8B/E2.R82G. The Irr.IgG1-derived clonal virus is designated CHIKwt.

**Table 3:** Amino acid variations in the E1 and E2 glycoproteins among clonal CHIKVs.

Clonal CHIKV	E1^a^			E2^b^		
	101	396	427	12	82	216
CHIK/5F #1,3, 4, 5 and 6	T	T	I	T	**G**	**E**
CHIK/5F #2	T	**A**	**T**	T	**G**	**E**
CHIK/8B #1,3, 4, 5 and 6	**M**	T	I	**I**	R	V
CHIK/8B #2	**M**	T	I	T	R	V
CHIK/5F+8B #1-6	T	T	I	T	**G**	V
CHIK/Irr #1-2	T	T	I	T	R	V

a,b Numbers refer to the *aa* positions within the E1 and E2 CHIKV glycoproteins, respectively. The *aa* variations associated with the CHIKV-specific mAb are highlighted in bold.

**Figure 2:**
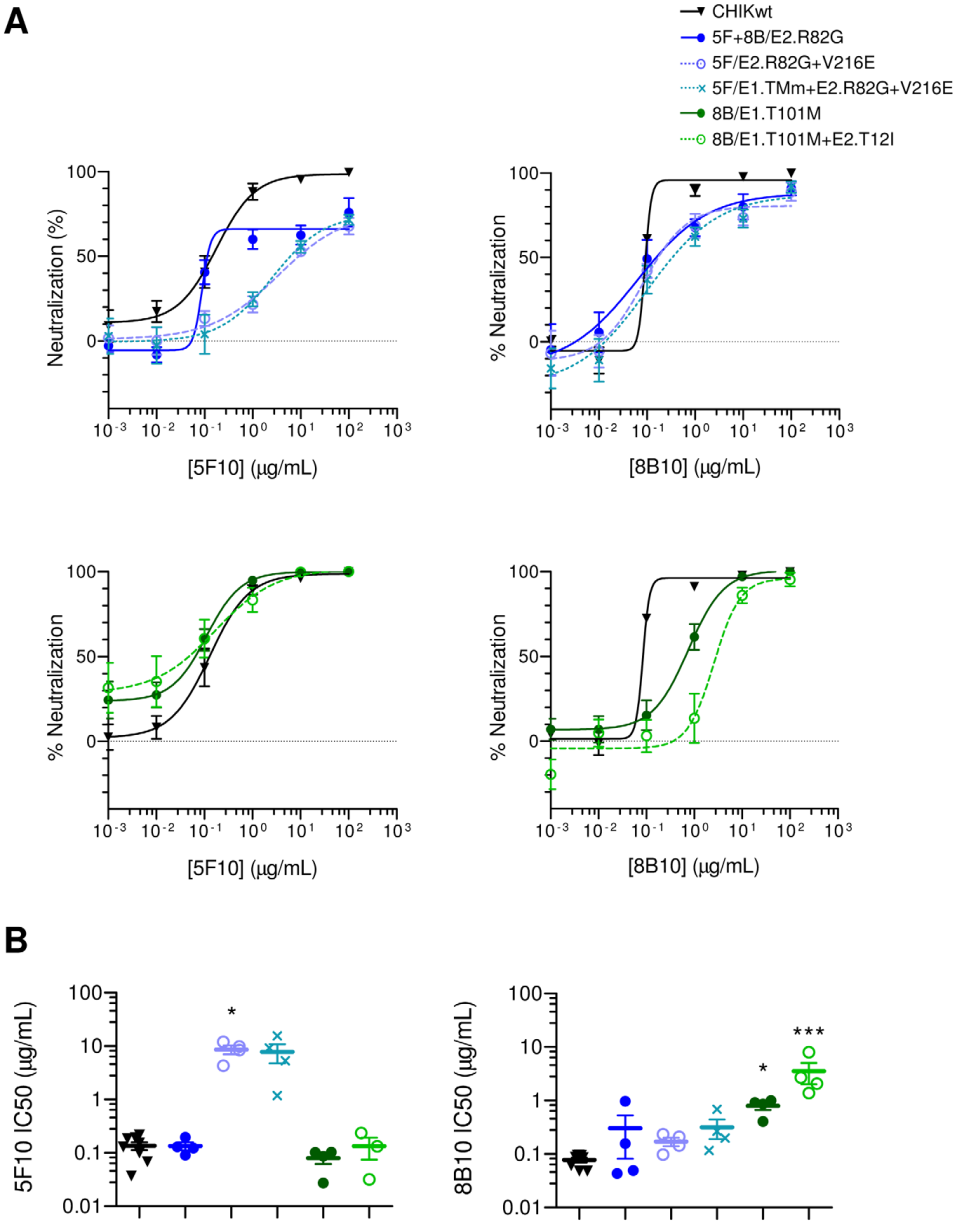
5F10 and 8B10 mAb neutralizing potency against clonal CHIKVs. CHIKV variants have been labelled according to their residue substitutions and corresponding mAb added to the culture medium during the serial passages. (**A**) Neutralizing capacities of the mAb against plaque-purified CHIKV were evaluated in PRNT over a concentration range of 1 ng-100 µg/ml. Shown are the mean and SEM from 4 independent experiments performed in duplicate with non-linear regression fitting curves. (**B**) The calculated IC50 from 3 or 4 independent experiments and mean ± SEM are shown (*P*-values were calculated using Kruskal-Wallis test and Dunn's post-test, and compare with the IC50 against CHIKwt: *, *p*<0.05; ***, *p*<0.001).

In PRNT assays, mAb 5F10 neutralized 5F/E2.R82G+V216E and 5F/E1.TMm+E2.R82G+V216E far less efficiently than CHIKwt (Figure 2). There were no significant differences between the two mutants, suggesting that neither of the two mutations in E1 had any effect on the neutralization potency of mAb 5F10. Although high concentrations of mAb 5F10 did not neutralize the CHIKV mutant containing E2.R82G alone (5F+8B/E2.R82G) as efficiently as CHIKwt, there was no significant difference in IC50 between 5F+8B/E2.R82G and CHIKwt, suggesting only a minimal effect of the mutation E2.R82G on 5F10-dependent CHIKV neutralization. Together, these data suggest a key role for the E2.V216E mutation in mediating CHIKV resistance to 5F10-dependent neutralization.

In parallel, mAb 8B10 neutralized 8B/E1.T101M less efficiently than CHIKwt (Figure 2), indicating a contribution for the mutation E1.T101M in mediating CHIKV resistance to 8B10-dependent neutralization. However, the viral mutant 8B/E1.T101M was more efficiently neutralized by mAb 8B10 than the dual mutant 8B/E1.T101M+E2.T12I (Figure 2), demonstrating that both the E1.T101M and E2.T12I mutations confer CHIKV resistance to 8B10-dependent neutralization.

Interestingly, the 8B10-derived CHIKV mutants remained efficiently neutralized by mAb 5F10, and *vice versa* (Figure 2). Thus, the binding sites of 5F10 and 8B10 are most likely different.

As the two mutations in the E1 trans-membrane domain were not associated with resistance to mAb-dependent neutralization, 5F/E1.TMm+E2.R82G+V216E was excluded from further analyses. Although the E2.R82G mutation was not found to be associated with significant resistance to mAb-dependent neutralization in PRNT assay, as this was the only mutation to be selected under dual treatment with mAb 5F10 and 8B10 (Tables 1 and 3), It was hypothesized that this mutation might be associated with a CHIKV immune evasion mechanism which was not detectable by PRNT assay. We therefore retained the 5F+8B/E2.R82G mutant for analysis in subsequent experiments.

### E2.V216E and E1.T101M abolish the binding of 5F10 and 8B10 to CHIKV

**Figure 3:**
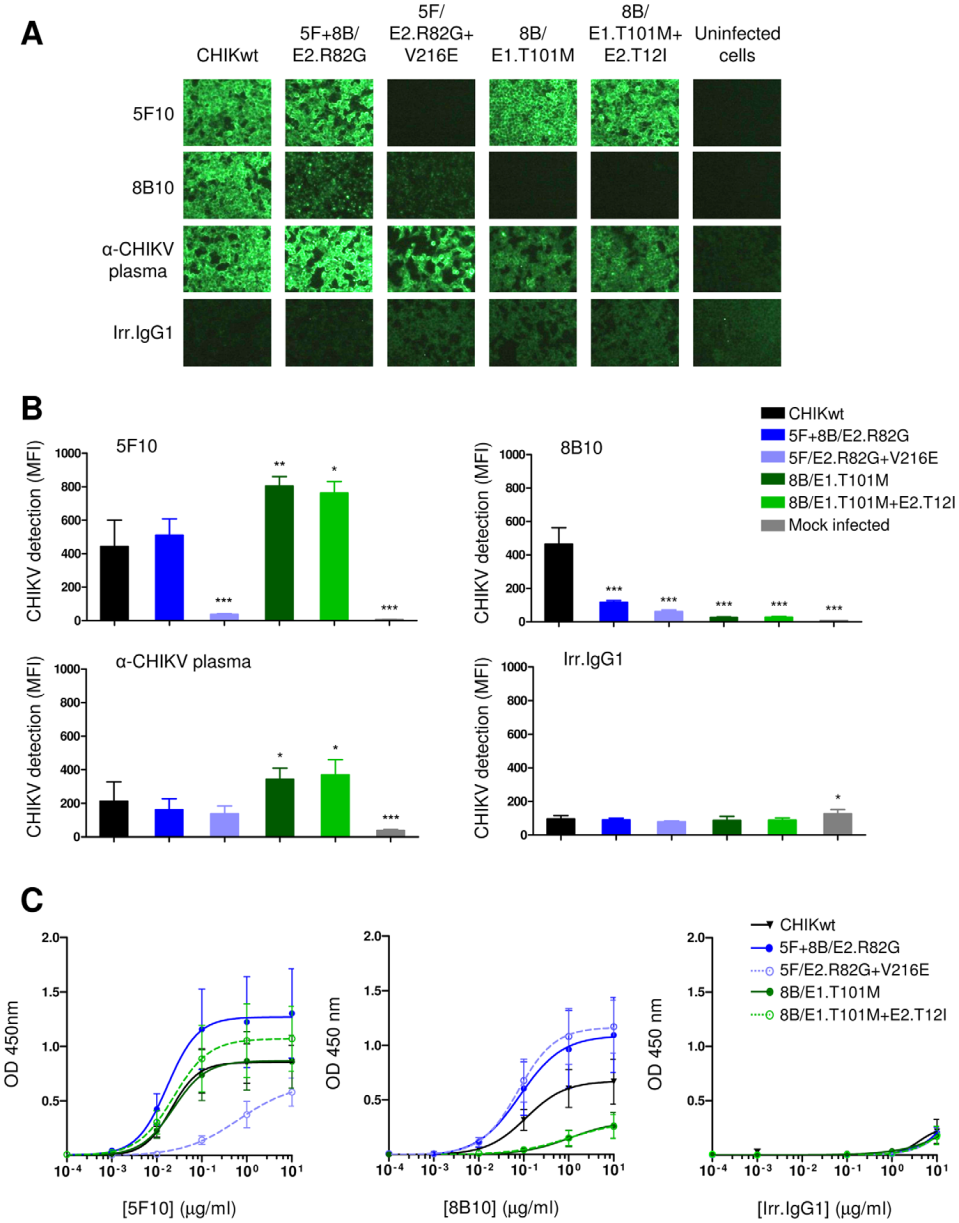
Analysis of 5F10 and 8B10 mAb binding to clonal CHIKVs. (**A**) Analysis of mAb binding to CHIKV-infected cells by immunofluorescence assay. HEK293T cells either non-infected, or infected with the indicated CHIKVs, were probed with mAb 5F10, 8B10, anti-CHIKV plasma, or with irrelevant IgG1. Images were captured at 100× magnification. (**B**) Quantitative analysis of mAb binding to CHIKV-infected cells by Cellomics ArrayScan. HEK293T cells, either mock infected or infected with the indicated CHIKVs, were probed with mAb 5F10, 8B10, anti-CHIKV plasma, or with irrelevant IgG1. Images were captured at 10× magnification. Displayed are mean fluorescent intensity (MFI) and SEM from 3 independent experiments performed in quadruplicate (*P*-values were calculated by Kruskal-Wallis test and Dunn's post-test, and compare with the MFI of CHIKwt-infected cells: *, *p*<0.05; **, *p*<0.01; ***, *p*<0.001). (**C**) ELISA analysis of mAb binding to plaque-purified CHIKV particles. ELISA plates were coated with 10^4^ UV-inactivated CHIKV particles, prior to being incubated with mAb (0.1 ng-100 µg/mL). Bound mAb were detected using HRP-conjugated goat anti-human IgG and TMB substrate. The OD was measured at 450 nm. Shown are mean and SEM from 3 independent experiments performed in duplicate.

To clarify the mechanism(s) associated with the neutralization escape mutations, we next analyzed the capacity of mAb 5F10 and 8B10 to bind to clonal CHIKVs (Figure 3).

Binding tests performed on CHIKV-infected cells (Figure 3A and 3B) and on CHIKV particles (Figure 3C) showed that the binding of 5F10 to 5F/E2.R82G+V216E was drastically impaired, whereas 5F10 efficiently bound the single mutant 5F+8B/E2.R82G. The binding of 8B10 to both 8B/E1.T101M and 8B/E1.T101M+E2.T12I was severely impeded. Interestingly, 5F10-derived mutated viral particles remained bound by 8B10 and *vice versa* (Figure 3C), confirming that mAb 5F10 and 8B10 exhibit distinct epitope specificities. However, binding assays performed on infected cells showed that 5F10-derived viruses were less efficiently bound by 8B10 when compared with CHIKwt (Figure 3A and 3B). These data are consistent with our previous suggestion that the target epitope for 8B10 was likely to be conformation-dependent [28]. It is therefore possible that, while expressed in infected cells and under experimental conditions used for the binding test on infected cells, the 8B10 epitope is indirectly modified by substitution of distantly located residues, such as E2.82 or E2.216.

Taken together, these data demonstrate that mutations E2.V216E and E1.T101M abolish the binding of mAb 5F10 and 8B10 to CHIKV, respectively.

### Structural location of key amino acid residues in the alphavirus E1/E2 glycoprotein

**Figure 4:**
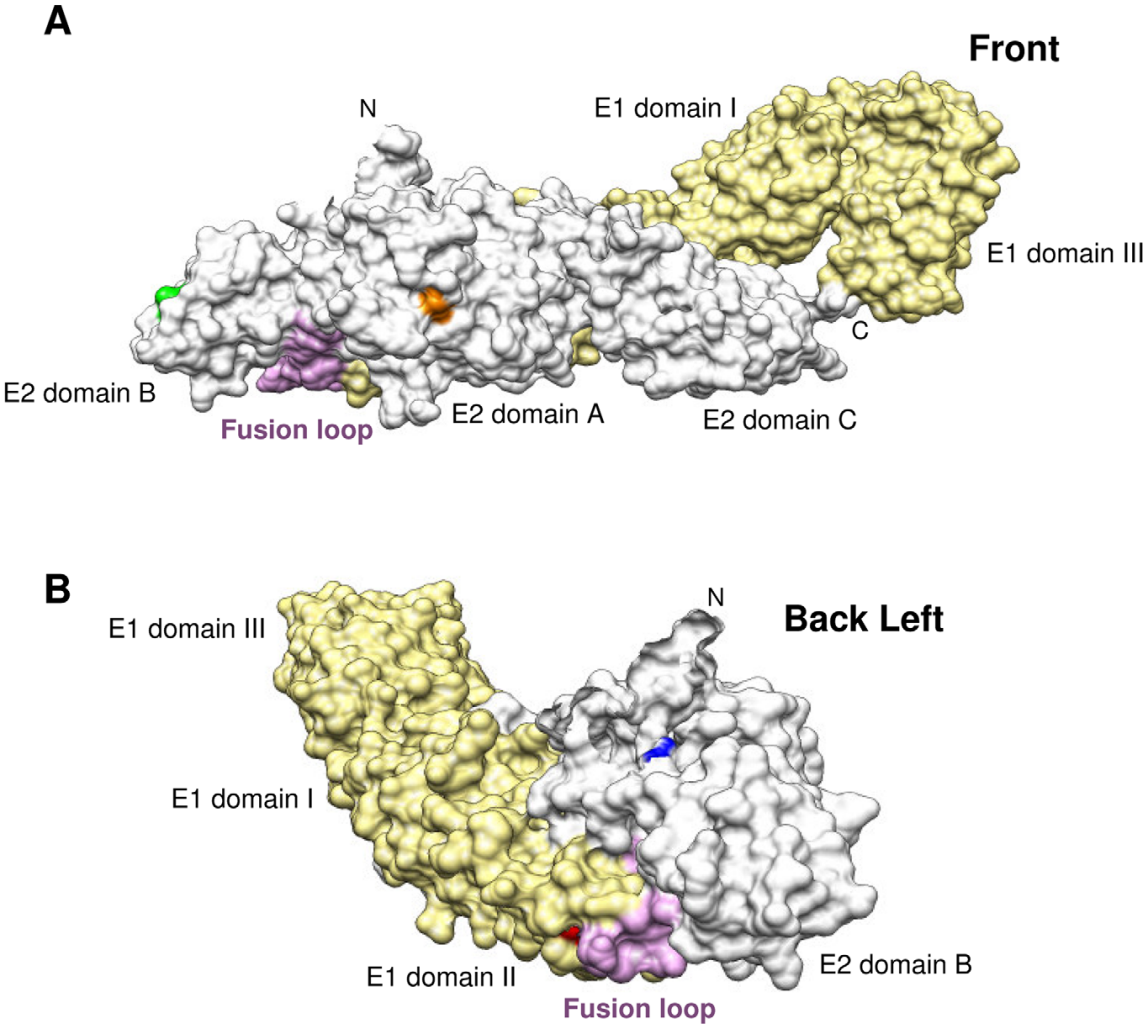
Location of E1.101, E2.12, E2.82 and E2.216 in the CHIKV E1/E2 heterodimer. Based on structural data retrieved from protein database records, the 3D organization of E1 (pale yellow), E2 (white), and the E1 fusion loop (pink) are shown for 3N44 under neutral pH conditions. (**A**) Front view of E2 with the location of E2.216 (green) and E2.82 (orange). (**B**) Back left view of E2 and E1 with the location of E2.12 (blue) and E1.101 (red).

To further clarify the CHIKV domains involved in virus interaction with mAb 5F10 and 8B10, we used Chimera software to locate residues E1.101, E2.12, E2.82 and E2.216 within the CHIKV E1/E2 heterodimer for which the crystal structure was recently resolved under neutral pH conditions [22] (Figure 4).

The E2.82 residue is exposed at the surface of the E2 domain A (Figure 4A), while the E2.216 residue is found at the tip of the E2 domain B (Figure 4A), in a position easily accessible to antibody binding.

The E1.101 residue is located within the E1 domain II next to the fusion loop (Figure 4B), suggesting that mAb 8B10 might target the fusion peptide itself. However, under neutral pH conditions, the exposed surface of the native viral particle is largely comprised of the E2 domains A and B, whereas the E1 protein remains concealed beneath the E2 protein, and its fusion peptide fits into a “groove” which is delineated by E2 domains A and B [22]. Therefore, under neutral pH conditions, E1.101 is unlikely to be accessible to antibody binding.

The E2.12 residue, which is involved in resistance to 8B10-dependent neutralization together with E1.101 (Figure 2), is located within the E2 domain A, directly above the “groove” which incorporates E1.101 (Figure 4B).

**Figure 5:**
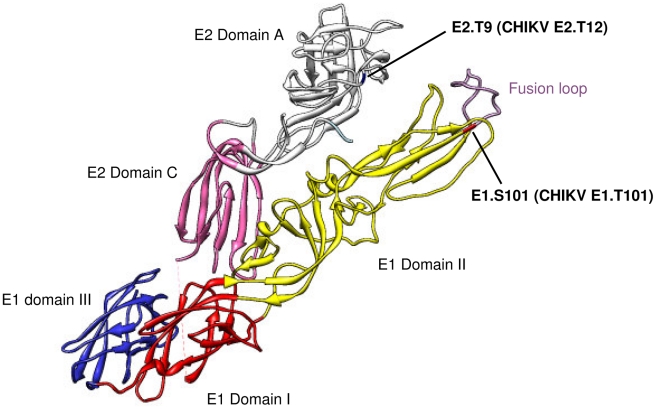
Location of Sindbis virus residues E1.S101 (CHIKV E1.T101) and E2.T9 (CHIKV E2.T12) in the E1/E2 heterodimer. Indicated residues were located based on structural data retrieved from protein data base records (3MUU, under acidic pH conditions).

Noteworthy, under acidic pH conditions, the alphavirus domain B becomes disordered and releases the fusion loop [23]. Interestingly, on the E2/E1 spatial arrangement of Sindbis virus (closely related with CHIKV) resolved under acidic pH conditions [23], the residues E1.101 and E2.12 are located on the internal side of the opened “groove”, facing each other (Figure 5). This suggests that after virus internalization into host cell and within the acidic endosomal compartment, the residues E1.T101 and E2.T12 (corresponding to Sindbis virus E1.S101 and E2.T9) may become more accessible to antibodies and come together to form a transitional epitope.

Taken together, these structural data strongly suggest that the 5F10 epitope is located at the tip of the CHIKV E2 domain B which contains the E2.216 residue. While the 8B10 target antigen remains somewhat ambiguous, our data suggest that this antibody may recognize a transitional epitope closely associated with the CHIKV fusion peptide.

### Fitness characteristics of the CHIKV E2.R82G mutant suggest viral cell-to-cell transmission

The alignment of available CHIKV E1 and E2 protein sequences obtained from GeneBank (data not shown) indicated that all published CHIKV variants contain the residues E1.T101, E2.T12, E2.V216 and E2.G82, except for one strain: TSI-GSD-218, which contains E2.I12 and E2.R82. Thus, among the mutations selected here, E1.T101M, E2.T12I and E2.V216E clearly modify highly conserved CHIKV residues, while E2.R82G instead restores a highly conserved viral residue. These data indicate that the CHIKV11 isolate used in the current study is atypical with regards to the E2.82 residue, and suggest an important role for the E1.T101, E2.T12, E2.G82 and E2.V216 residues in the CHIKV life cycle. Therefore, the substitution of these conserved residues was expected to modify CHIKV fitness, and thus, we next investigated fitness characteristics of 5F10/8B10-resistant CHIKV escape mutants.

**Figure 6:**
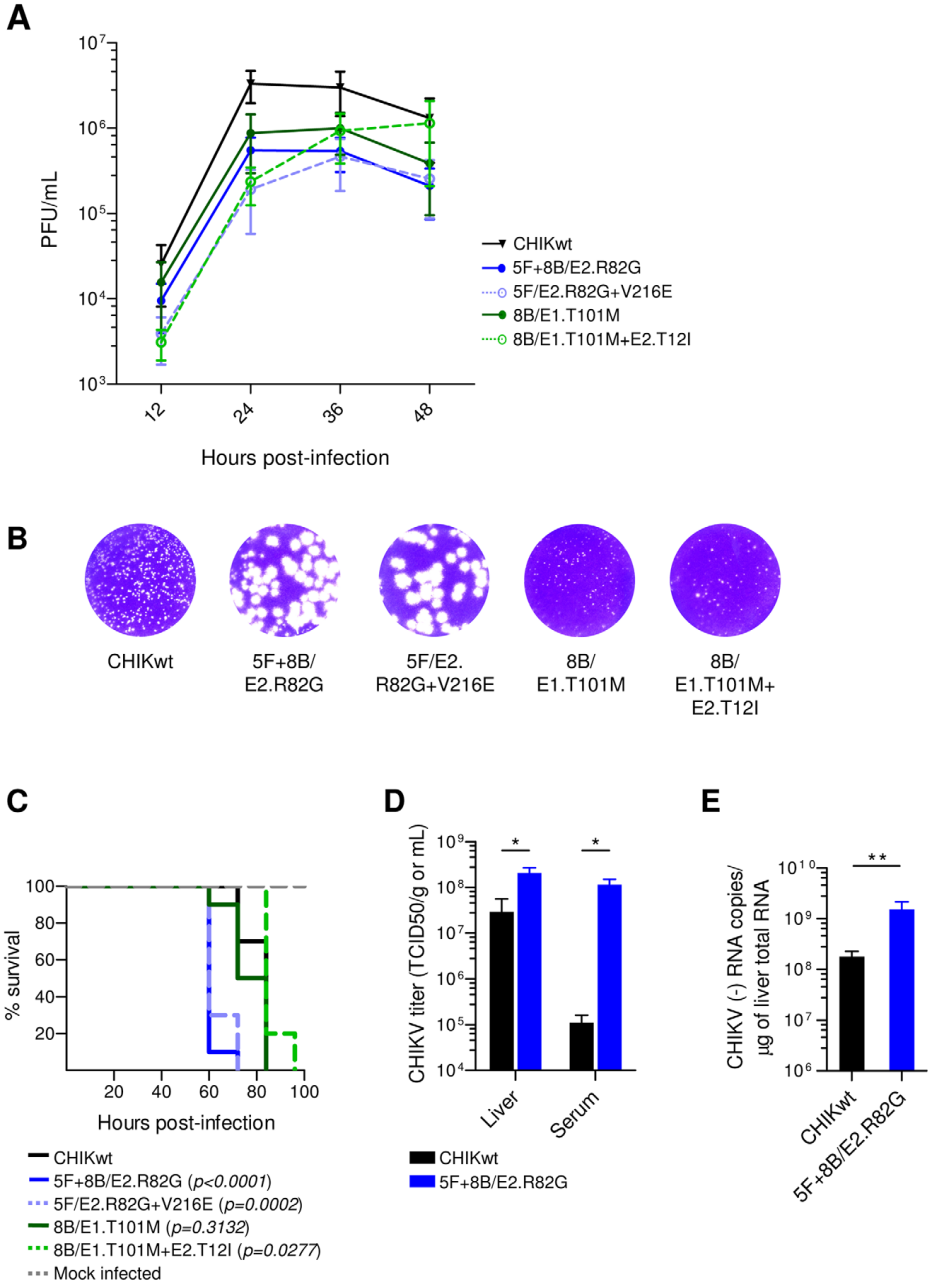
Viral fitness of CHIKV neutralization-escape mutants. (**A**) Vero cells were infected with plaque-purified CHIKV (MOI = 0.1). The number of PFU within the supernatants was determined by Plaque Assay at various times post-infection. Shown are mean and SEM from 3 independent experiments performed in duplicate. (**B**) Shown are typical plaque patterns by crystal violet staining at 12 h post-infection. (**C**) AGR129 mice were inoculated with plaque-purified CHIKV (10^3^ PFU). PBS-inoculated mice were used as negative controls. Mice were observed every 12 h to determine post-infection survival. Shown are the survival curves derived from 3 independent experiments (CHIKV, n = 10; Mock infected, n = 6). *P*-values were determined by Grehan-Breslow-Wilcoxon test and compare CHIKV mutants with CHIKwt. (**D**) Mice were inoculated with CHIKwt or 5F+8B/E2.R82G (10^3^ PFU) and the CHIKV load in serum and liver was quantified 48 h post-infection by TCID50. Shown are mean and SEM for 5 mice per group. *P*-values were determined using Mann-Whitney test (*, *p*<0.05). (**E**) At 48 h post-infection, the amount of CHIKV (-)RNA in liver was determined by quantitative RT-PCR. Shown are mean and SEM for 5 mice per group. *P*-values were determined using Mann-Whitney test (**, *p*<0.01).

We first assessed the *in vitro* extra-cellular spreading of the mAb-resistant CHIKV mutants. Vero cells were infected with clonal CHIKVs, and viral titer was next determined by Plaque Assay and expressed as the number of Plaque Forming Units (PFU) per mL of cell culture supernatant. Over the 48 h growth period, all CHIKV escape mutants led to significant decreases in PFU number, when compared with CHIKwt (Figure 6A). These results demonstrate that CHIKV variants which escape 5F10- and/or 8B10-dependent neutralization are impaired in extra-cellular viral spreading *in vitro*.

We next investigated the *in vivo* fitness of the CHIKV neutralization-escape mutants. AGR129 immuno-compromised mice [29] were used to determine survival after infection with the different clonal CHIKVs (Figure 6C). Mice infected with 8B/E1.T101M were indistinguishable from control mice infected with CHIKwt, suggesting that the E1.T101M mutation does not interfere with CHIKV fitness *in vivo*. In contrast, death among mice infected with 8B/E1.T101M+E2.T12I was delayed, when compared with mice infected with CHIKwt, suggesting that the E2.T12I mutation impairs CHIKV fitness *in vivo*. Interestingly, mice infected with 5F+8B/E2.R82G or 5F/E2.R82G+V216E died significantly earlier than those infected with other CHIKV variants. However, no difference was observed between mice infected with either 5F+8B/E2.R82G or 5F/E2.R82G+E2.V216E, suggesting that the E2.R82G mutation is entirely responsible for the rapid post-infection mortality, and that the E2.V216E mutation does not alter *in vivo* CHIKV fitness.

Mice were next infected with either CHIKwt or 5F+8B/E2.R82G and viral load was determined in serum and liver at 48 h post-infection (Figure 6D), alongside quantification of CHIKV (-) RNA in liver (Figure 6E). In both liver and serum, viral load of 5F+8B/E2.R82G was significantly higher than that of CHIKwt, with the largest difference (∼1000-fold) being observed in serum (Figure 6D). Likewise, the number of 5F+8B/E2.R82G (-) RNA copies in liver was ∼10-fold higher than for CHIKwt (Figure 6E). These data demonstrated that 48 h post-infection, the level of 5F+8B/E2.R82G replication was higher than that of CHIKwt. Moreover, a previous study in an alternative murine model of immunodeficiency reported that CHIKV is detected in the liver prior to being detected in the serum [30]. Therefore, our data suggest that 5F+8B/E2.R82G spreads faster *in vivo* than CHIKwt, since CHIKwt load was reduced in serum compared with liver, while the 5F+8B/E2.R82G load was comparable in both tissues (Figure 6D). The enhanced *in vivo* fitness of 5F+8B/E2.R82G supports an important role for the E2.G82 residue in CHIKV life cycle, which may explain the high conservation of this residue among CHIKV strains described as of now.

Since measuring the relative size of virus-induced plaques is commonly performed to monitor viral cell-to-cell transfer [31]–[33], we were intrigued to observe that 5F+8B/E2.R82G and 5F/E2.R82G+V216E gave rise to bigger plaques compared with alternative CHIKV variants (Figure 6B). This “big plaques” phenotype, together with the impaired *in vitro* extra-cellular viral spreading (Figure 6A), suggested that CHIKV might disseminate directly from cell to cell, in a manner induced or enhanced by the mutation E2.R82G. Interestingly, the rapid *in vivo* spreading of 5F+8B/E2.R82G when compared with CHIKwt (Figure 6C–6E), further suggested an E2.R82G-associated CHIKV cell-to-cell transmission, as this mode of dissemination is considered to be faster than extra-cellular transmission [34].

### CHIKV cell-to-cell transmission is enhanced by E2.R82G

**Figure 7:**
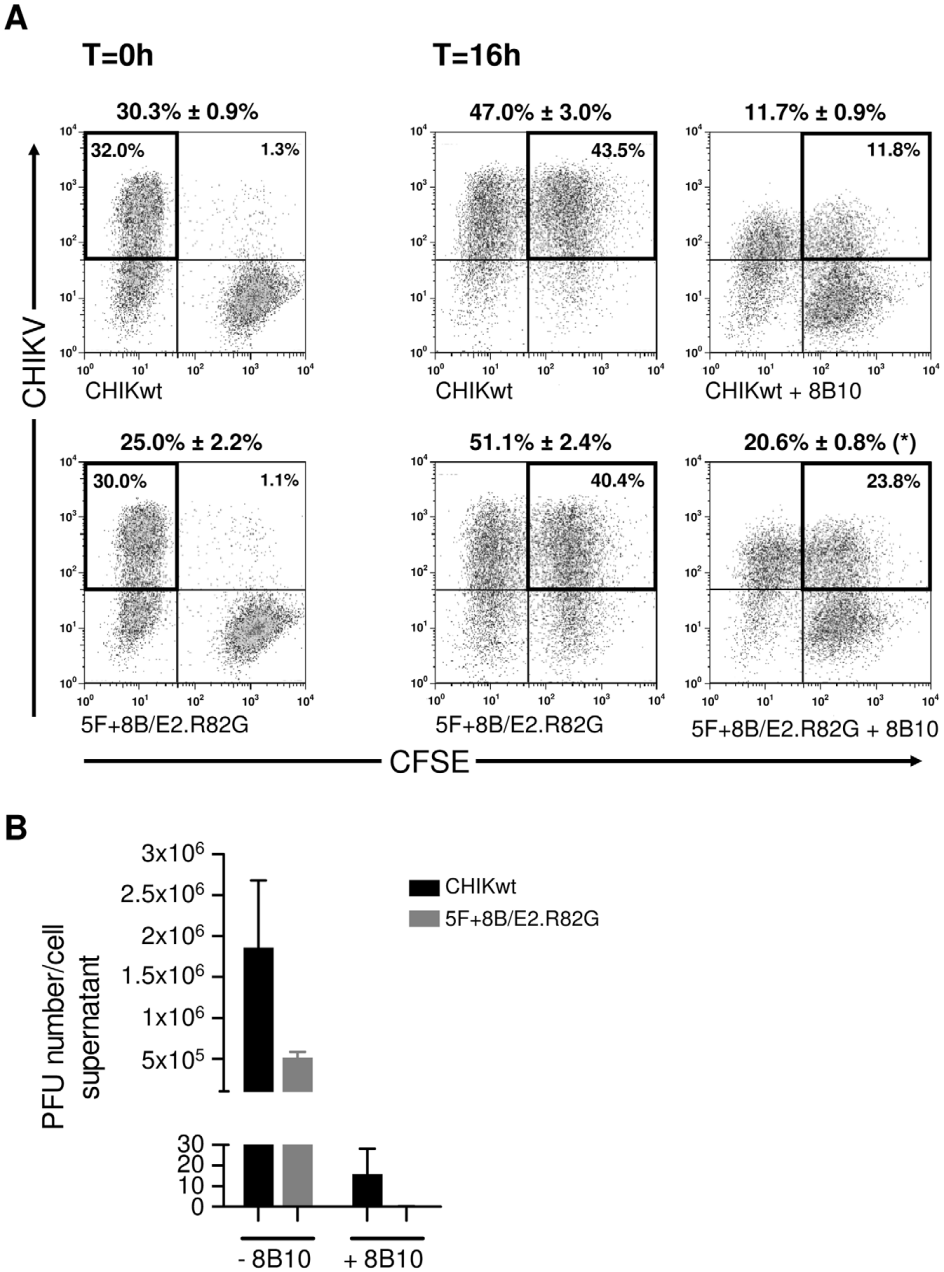
Quantification of CHIKV cell-to-cell transmission. CHIKV-infected HEK293T cells (producer cells) were co-cultured with CFSE-labeled naïve HEK293T cells (target cells) in the absence or presence of mAb 8B10. (**A**) The number of infected cells was quantified by flow-cytometry either immediately (T = 0 h), or after 16 h of co-culture (T = 16 h). Above each panel are shown the mean number of infected producer (T = 0 h) or target (T = 16 h) cells alongside the SEM (5 independent experiments performed in duplicate). *P*-values were determined using Wilcoxon test, and compare CHIKwt and 5F+8B/E2.R82G infectivity (*, *p*<0.05). (**B**) After 16 h of co-culture, the number of extra-cellular CHIKV particles within the supernatants was determined by Plaque Assay. Shown are mean and SEM from 5 independent experiments performed in duplicate.

Direct cell-to-cell transmission was previously proposed to occur during CHIKV infection *in vitro* [35], but formal demonstration of this mode of dissemination has been lacking. To address this possibility, unlabeled HEK293T cells were infected with either CHIKwt or 5F+8B/E2.R82G. Ten hours post-infection, the infected cells (producer cells) were co-cultured with CFSE-labeled non-infected HEK293T cells (target cells), in the presence or absence of mAb 8B10. After 0 h and 16 h of co-culture, the number of CHIKV-infected cells was determined by flow-cytometry (Figure 7A). By 16 h, the majority of target cells (47.0% and 51.1% for CHIKwt and 5F+8B/E2.R82G, respectively) were CHIKV-infected when cultured without mAb 8B10. Interestingly, even in the presence of 8B10 neutralizing pressure, the mean proportion of infected target cells was 11.7% for CHIKwt and 20.6% for 5F+8B/E2.R82G. We therefore hypothesized that these cells had either been infected by direct CHIKV cell-to-cell transmission, or by 8B10-resistant extra-cellular CHIKV particles. However, in the presence of mAb 8B10, extra-cellular CHIKV particles were undetectable in the 5F+8B/E2.R82G-derived supernatants, and only an extremely low titer was measured in CHIKwt-derived supernatants (Figure 7B), demonstrating that, under 8B10 neutralizing pressure, target cells infection resulted from CHIKV direct cell-to-cell transmission. Moreover, the significant higher percentage of infected target cells detected with 5F+8B/E2.R82G as compared with CHIKwt indicates that CHIKV cell-to-cell transmission is enhanced by the E2.R82G mutation.

**Figure 8:**
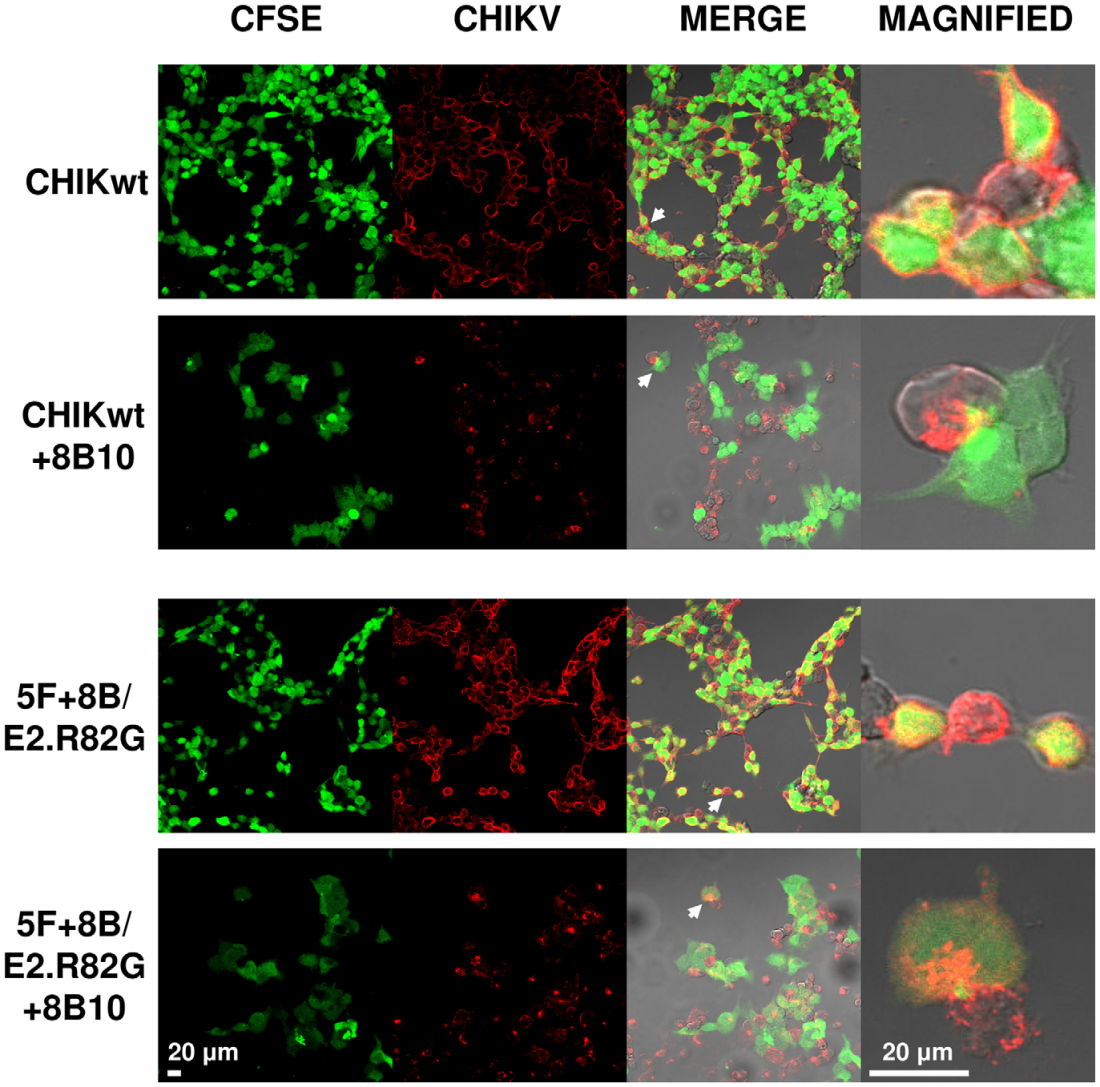
CHIKV is polarized to sites of cell-cell contact under neutralizing antibody pressure. CHIKV-infected HEK293T cells were co-cultured with CFSE-labeled naïve HEK293T cells (green) in the absence or presence of mAb 8B10. After 16 h of co-culture the cells were fixed, permeabilized, and stained for alphavirus expression (Alexa 647, red). Newly infected target cells appear orange. Magnification: ×40 or ×80.

To further confirm direct cell-to-cell transmission of CHIKV, some co-cultures were visualized by confocal microscopy. For both CHIKwt and 5F+8B/E2.R82G, in the absence of mAb 8B10, CHIKV staining was detected uniformly on the cell surface, whereas, under 8B10 neutralizing pressure, CHIKV staining was strongly polarized and virus was often detected in areas of cell-cell contact (Figure 8). These data strongly suggest that, when the virus is subjected to antibody dependent-neutralizing pressure, CHIKV dissemination occurs by direct cell-to-cell transfer at areas of cell membrane contact. Although these results further show that CHIKwt can also disseminate by direct cell-to-cell transfer, as less CHIKV-specific staining was detected in target cells with CHIKwt, when compared with 5F+8B/E2.R82G, these data confirm that the E2.R82G mutation enhances CHIKV cell-to-cell transmission.

## Discussion

We previously demonstrated potent *in vitro* neutralization of CHIKV using human mAbs 5F10 and 8B10 [28]. In the current report, we have characterized CHIKV variants that escape 5F10 and/or 8B10-dependent neutralization to identify the CHIKV E2 domain B and the fusion loop “groove” as the primary determinants of CHIKV interaction with these neutralizing antibodies. We have also demonstrated CHIKV cell-to-cell transmission, which involves the E2 domain A, and may represent a route by which the virus evades antibody-dependent neutralization.

Escape mutants have previously been described for three members of the alphavirus family; Sindbis virus [19], [36]–[38], Ross River virus [39] and Venezuelan Equine Encephalitis virus [40]–[42]. Several neutralization escape mutations have been identified within the alphavirus E2 domain A [37], [40] and domain B [19], [39], [40], as well as within the E1 domain II [40]. However, CHIKV mutants that escape antibody-dependent neutralization have not previously been reported, and the alphavirus residues E1.101 and E2.12 were not previously shown to be determinants of neutralizing antibodies binding. Consistent with the current report, the E2.216 residue has previously been identified as being involved in major B-cell neutralization epitopes for Sindbis virus, Ross River virus and Venezuelan Equine Encephalitis virus [19], [39], [40], suggesting that the region covering the E2.216 residue within the E2 domain B is an important antigenic domain shared by several alphaviruses. Of note, Coffey and Vignuzzi recently described mutations that affect the CHIKV E2 residues 229–234 as being due to selective pressure from neutralization [43]. These mutations affected the C-terminal end of the E2 domain B, as well as the ß-ribbon connector which links the domain B to the domains A and C [22], [23]. Although these mutations were not formally shown to be responsible for CHIKV resistance to neutralization [43], their location is consistent with a crucial role for the CHIKV E2 domain B in virus-neutralizing antibody interactions.

Our results strongly suggest that the 5F10 mAb epitope is located at the tip of the CHIKV E2 domain B, while 8B10 might recognize a transitional epitope, close to the fusion loop, which is likely to be exposed under acidic pH conditions. Interestingly, in addition to the disordering of the alphavirus envelope at acidic pH, virus binding to a host cellular receptor is believed to induce pH-independent shifting of the E2 domain B, leading to exposure of transitional epitopes prior to virion internalization [22], [23], [36], [44]. We therefore speculate that 8B10 mAb epitope might be accessible to antibody binding before virus internalization into host cell but upon CHIKV binding to a cellular receptor.

Based on their proposed epitope specificity, we speculate that 5F10 and 8B10 inhibit viral entry and fusion to the cell membrane, respectively. However, it has also been suggested that antibodies which target the E2 domain B might also affect the viral-cell fusion step, possibly by inhibiting domain disordering and fusion loop exposure [23]. Thus 5F10 might alternatively inhibit virus-cell membrane fusion instead of overtly inhibiting viral entry.

Although RNA viruses are usually prone to high nucleotide sequence evolution due to their lack of a proofreading polymerase [45]–[47], arboviruses seem less likely to genome modifications, as evidenced by their high rate of nucleotide sequence conservation over the time. This may be due to their lifecycle requirement to replicate in two taxonomically distinct hosts [48]–[50]. Thus, most mutations which occur during arboviruses replication appear to impair viral fitness. In line with these observations, we showed that modification of the conserved CHIKV residues E1.T101, E2.T12, E2.G82, and E2.V216 is associated with reduced viral fitness *in vitro* and/or *in vivo*.

After 8 cycles of neutralization/amplification, only partial CHIKV resistance to mAb 8B10 or 5F10+8B10 was observed. We then performed 5 additional rounds of neutralization/amplification while using increasing concentrations of mAb. However, even after 13 neutralization/amplification rounds under mAb selective pressure, we did not manage to select a CHIKV population fully resistant to 8B10 or 5F10+8B10 (data not shown). It is likely therefore that the mutation(s) required to fully escape 8B10 and 5F10+8B10 mAb give(s) rise to viruses that are unable to replicate robustly.

Cell-to-cell virus transmission is faster than extra-cellular spreading and enables viruses to evade the immune response [31], [34], [51]. Herpesviruses, flaviviruses, paramyxoviruses, poxviruses, retroviruses and rhabdoviruses have already been shown to employ this cell-associated infection route, either exclusively, or in parallel with extra-cellular viral dissemination [32], [34]. Cell-to-cell transmission seems to be limited to enveloped viruses which exit at the plasma membrane either by budding or by exocytosis, whereas lytic viruses disseminate only by extra-cellular transmission [32], [34]. Despite their biological and structural characteristics making them prone to cell-associated dissemination, alphaviruses have not previously been shown to transfer directly between cells. Interestingly, in an earlier report, direct CHIKV transmission between cells was postulated as a mechanism of antibody escape since the virus could be detected in cultured cells despite the presence of anti-CHIKV serum [35]. However, the presence of newly-infected cells was not demonstrated.

We demonstrate in this report, for the first time, CHIKV direct cell-to-cell transmission, and further show that this mode of dissemination is enhanced by the E2.R82G mutation. Interestingly, as the alignment of available CHIKV E2 protein sequences obtained from GeneBank (data not shown) revealed that the majority of CHIKV variants contain the residue E2.G82 (and not E2.R82), we speculate that direct cell-to-cell transmission is commonly used by CHIKV to disseminate in the presence of extra-cellular neutralizing antibodies. However, this remains to be shown with CHIKV isolates containing the residue E2.G82.

Viruses have evolved various mechanisms to disseminate from cell to cell. Pre-existing cell-cell contacts may be exploited, or virus-induced new contacts can be established between infected and uninfected target cells. [32], [34], [52]. The confocal microscopy images presented in the current report suggest that CHIKV may use pre-existing cell-cell contacts, possibly tight junctions, to transfer directly between cells. Further studies are now warranted to precisely characterize the mechanism of CHIKV cell-to-cell transmission.

The fact that CHIKV cell-to-cell transmission is enhanced by E2.R82G, suggests the involvement of the CHIKV E2 domain A in this mode of dissemination. Interestingly, the E2 domain A of both Venezuelan Equine Encephalitis virus and Sindbis virus has previously been shown to contain residues that are important for virus binding to cells, notably to heparan sulfate located at the cell surface [18], [53]–[55]. In this context, we speculate that the mutation E2.R82G may similarly enhance CHIKV binding to the target cell.

For the first time, we have identified CHIKV envelope domains that are recognized by human neutralizing immune responses, and we have been able to demonstrate direct cell-to-cell transmission of CHIKV. This mode of dissemination, which protects CHIKV from neutralizing host antibodies, might play an important role in establishment of CHIKV persistence. These findings advance our understanding of CHIKV-human host interactions and will aid the rational design of future domain-based vaccines against CHIKV, as well as inform further studies of CHIKV pathogenesis.

## Materials and Methods

### Ethics statement

This study was carried out in strict accordance with the guidelines of the Agri-Food and Veterinary Authority (AVA) and the National Advisory Committee for Laboratory Animal Research (NACLAR) of Singapore. All animal procedures were approved by the Institutional Animal Care and Use Committee (IACUC) of Biological Research Center, Biomedical Sciences Institutes, A*STAR, Singapore (IACUC number: #100515).

### Cells, antibodies and viruses

Vero cells (ATTC CCL-81) and HEK293T cells (ATCC CRL-N268) were cultured in DMEM-10% FCS (Gibco-Invitrogen). The CHIKV-neutralizing human mAb 5F10 and 8B10, the irrelevant human mAb HA4 (referred as Irr.IgG1), and the CHIKV isolate CHK/Singapore/11/2008 (referred as CHIKV11) have been described previously [28].

### Selection of 5F10 and 8B10-resistant CHIKV mutants

CHIKV11 (200 PFU) was incubated for 1 h at 37°C with 100 ng/ml 5F10 and/or 8B10 in DMEM-10% FCS. HEK293T cells were then incubated at 37°C for 1.5 h with mAb/CHIKV mixtures, prior to being further cultured in DMEM-10% FCS supplemented with additional mAb as before for 2 days. Cell supernatants were then collected and their infectious viral titer was determined by Plaque Assay; 200 PFU of rescued virus was subjected to a second round of neutralization/amplification in the presence of the same antibodies as employed in previous steps. Eight neutralization/amplification rounds were performed in total.

### CHIKV cloning

Vero cells were infected for 1.5 h with CHIKV (10 PFU/well) before being washed with PBS and cultured in DMEM-0.25% agarose for 2 days. Individual CHIKV colonies were then selected through the agarose layer and amplified separately in Vero cells. Plaque-purified clonal CHIKV genomes were then sequenced.

### Viral sequencing

Viral RNA was extracted from 140 µl supernatant from CHIKV infected-cells using the QIAamp Viral RNA Mini kit (Qiagen). For each viral RNA, two independent full-length cDNAs were synthesized using random hexamers and SuperScript III First-Strand kit (both from Invitrogen). Purified cDNAs were PCR-amplified using Taq PCR Master Mix Kit (Qiagen) and several primer pairs designed to cover the C-E1 CHIKV polyprotein encoding sequence and to generate ∼1000 bp-long overlapping PCR fragments. PCR fragments were sequenced (Aitbiotech) and results were analyzed using Lasergene 7 software.

### Next-Generation sequencing

A 200 bp cDNA library was synthesized from 56 ng of extracted CHIK/Irr RNA using the mRNA-seq Sample Prep Kit (Illumina) according to the manufacturer's instructions. The cDNA library was then sequenced using the Illumina GAIIX genome analyzer (Next Generation Sequencing Core facility, Genomic Institute of Singapore) at the coverage of 67436×. Unique reads were subsequently aligned with the consensus sequence encoding CHIKV11 structural proteins (C-E1) using the Burrows-Wheeler aligner, and site-specific nucleotide frequencies were determined using SAMtools Pileup.

### Plaque Reduction Neutralization test and mAb potency

The Plaque Reduction Neutralization test and determination of mAb potency were performed as previously described [28].

### Binding assays

Immunofluorescence Assay: HEK293T cells were infected with CHIKV and then fixed as previously described [28]. One µg/ml mAb or human anti-CHIKV polyclonal plasma (1∶200) was added to either CHIKV-infected or non-infected cells for 1 h at 37°C. Antibody binding was detected by addition of 2 µg/ml Alexa-488-labeled mouse anti-human IgG (Invitrogen) followed by fluorescence microscopy (NIKON ECLIPSE TS 100) at 100× magnification. Alternatively, mAb binding to CHIKV-infected cells was quantified using a Cellomics HCS Reader (Cellomics ArrayScan, Thermo Fisher Scientific): HEK293T cells were infected with CHIKV as previously described [28]. At 24 h post-infection, the cells were washed and fixed overnight with 4% paraformaldehyde. One µg/ml mAb or human anti-CHIKV polyclonal plasma (1∶200) was added to CHIKV-infected or non-infected cells for 1 h at 37°C. Antibody binding was detected by addition of 10 µg/ml Alexa-488-labeled mouse anti-human IgG (Invitrogen). Cell nuclei were visualized using DAPI staining and images were captured at 10× magnification.

In some experiments, 96-well plates were coated with UV-inactivated plaque-purified CHIKVs (10^4^ PFU/well) for analysis by ELISA. A range of mAb concentrations (0.1 ng-10 µg/mL) were added to the wells for 1 h at RT. Bound mAb were detected using HRP-conjugated goat anti-human IgG (Jackson ImmunoResearch), followed by incubation with 3,3′,5,5′-tetramethybenzidine substrate (Sigma). The reaction was stopped by addition of HCl (1 M) and absorbance was measured at 450 nm using the TECAN Infinite M200 Monochromator Microplate Reader (TECAN).

### *In vitro* viral fitness analysis

Vero cells were infected with plaque-purified CHIKV mutants (MOI = 0.1) for 1.5 h prior to being cultured in DMEM-10% FCS. The number of PFU in each supernatant was determined by Plaque Assay at various times post-infection.

### *In vivo* experiments

AGR129 mice (IFN-α/ß/γR^−/−/−^ and RAG-2 deficient, [29]) were used at 8–12 weeks of age and were inoculated intravenously with 10^3^ CHIKV PFU diluted in 200 µl PBS (or with PBS-alone for control mice). In survival experiments, mice were observed at 12 h intervals thereafter. For viral load quantification, mice were bled at 48 h post-infection, prior to sacrificing them and liver harvesting. Viral load in sera and homogenized liver supernatants were determined by measurement, using Vero cells, of tissue cytopathic infectious dose 50 (TCID50) expressed as TCID50/mL and TCID50/g, respectively. In parallel, total RNA was extracted from 1 g of liver using *TRI* reagent (Sigma) according to the manufacturer's instructions. To quantify CHIKV (-)RNA copies, 3.6 ng total RNA extracted from liver was subjected to qRT-PCR as previously described [56].

### Co-culture assays

HEK293T cells were infected with CHIKV for 1.5 h (MOI = 0.1), then washed and cultured in DMEM-10% FCS. At 10 h post-infection, naïve “target” HEK293T cells were labeled with 10 µM CFSE (Sigma) and 3×10^5^ labeled “target” cells were co-seeded into 12-well plates with 3×10^5^ extensively washed infected or non-infected “producer” cells. The cells were then co-cultured in DMEM-10% FCS, supplemented or not with 200 µg/mL 8B10. At 0 h and 16 h post-co-culture the cells were harvested, washed and fixed/permeabilized (BD Cytoperm/Cytofix, BD Biosciences), prior to intracellular staining with 5 µg/mL mouse IgG2a anti-alphavirus (3581) (Santa Cruz Biotechnology) followed addition of 4 µg/mL Alexa 647-conjugated goat anti-mouse IgG (Invitrogen). The proportion of infected cells was then determined by flow-cytometry (FACSCalibur, BD Biosciences). Cell supernatants were also collected in parallel after 16 h co-culture for analysis by Plaque Assay to quantify infectious extra-cellular CHIKV. Alternatively, at 14 h post-infection, 10^5^ HEK293T cells were seeded into μ-Slide 8 well plates (Ibidi) with 10^5^ CFSE-labeled uninfected cells and then cultured as described above. After 16 h of co-culture the cells were washed with PBS, fixed with PBS-4% paraformaldehyde and then permeabilized in PBS-0.5% Triton X-100. The permeabilized cells were then stained with 3 µg/mL mouse IgG2a anti-alphavirus (3581) followed by addition of 3 µg/mL Alexa 647-conjugated goat anti-mouse IgG (Invitrogen). ProLong Gold (Invitrogen) was added to the wells and fluorescence was analyzed using an Olympus FV1000 confocal microscope at ×40 magnification (or at ×80 in highlighted panels).

### Statistical analyses

Data were analyzed using GraphPad Prism 5. The specific statistical tests used are indicated in the respective figure legends.
